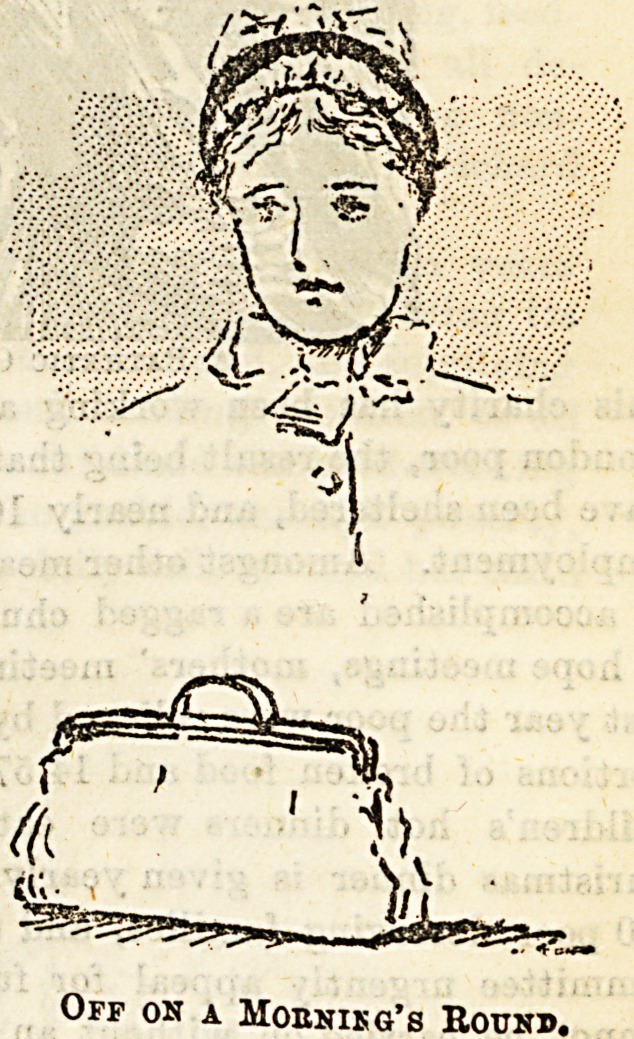# Nursing Institutions

**Published:** 1891-01-03

**Authors:** 


### NURSING INSTITUTIONS.

WE specially desire to commend to the generous consideration of the charitable the many nursing associations in various parts of London which provide nurses for the sick poor. There is no branch of work more worthy or deserving than this, and it is desirable that it should be encouraged by increased liberality on the part of the public. We hope that our readers may be induced to send contributions to one of the following :—

**Bible Woman Nurse,** 2, Adelphi Terrace, Strand, W.C. Hon. sec., G. Selfe Leonard.

**East London Nursing Society,** 49, Philpot Street, Commercial Road, E. Secretary, A. W. Lacey.

**Nursing Sisters of St. John the Divine,** 68 and 70, Drayton Gardens, South Kensington, S.W. Hon. sec., Lieut.-General J. E. T. Nicolls. Sister superior, Selina S. Wilson.

**St. Helena Home for Trained Nurses and Paying Patients,** 1, Grove End Road, N.W. Sec., Mr. W. H. Kidson. Lady Supt., Miss Robertson.

**Workhouse Infirmary Nursing Association,** of 6, Adam Street, Strand, is one of the best of our charitable nursing institutions. It has worked a perfect revolution in the workhouse infirmaries throughout the country, and the dying pauper is now sure of a certain amount of care and attention when his last trial has arrived, and he has to face the valley of the shadow of death. The association has fought its way nobly against strong prejudices; it has been a pioneer society, and therefore has had a hard path to travel, and it still needs support and increased funds. There yet remain infirmaries where the sick and dying are trusted to the tender mercies of imbecile paupers, and if only the association had larger funds this would soon be remedied. The association has an income of about £300 a-year, and the amount of work it does with this money is simply extraordinary. Hon. sec., Miss Wilson.

**Metropolitan and National Nursing Association,** 23, Bloomsbury Square, W.C.—An association whose object is to provide the sick poor with all the advantages of skilled attendance in their own homes, bringing into many houses not only the benefits derived directly from trained knowledge, but in many instances the first suggestions that such benefits were within the reach of all. To those who visit amongst our poor, the sight of these nurses in their neat bonnet and cloak, and carrying the inevitable black bag (the badge of their mission of usefulness) is as familiar as it is welcome. The following are the names of nursing homes founded by the Committee, or supplied with nurses trained at the Central Home, which will show what a wide scheme of usefulness this Association embraces: Holloway, Paddington, Battersea, Hampstead, Kensington, Newington and Walworth, Chelsea, Windsor, Egham, Westminster, Bishop Auckland, Hertford, Hereford, and Haggerston. During the past year 746 cases were nursed, and the number of visits paid was 19,900. Contributions should be sent to the honorary secretary, the Rev. Dacre Craven, 23, Bloomsbury Square, W.C. Lady superintendent, Miss Mansel.

OFF ON A MORNING'S ROUND.

**North London Nursing Association,** 413, Holloway Road, N. Hon. sec., Mr. C. A. Powell. Superintendent, Miss De Lüttischan.

## Figures and Tables

**Figure f1:**